# Analysis of the relationship between sleep-related disorders and cadmium in the US population

**DOI:** 10.3389/fpubh.2024.1476383

**Published:** 2024-10-25

**Authors:** Zhuanbo Luo, Ning Zhu, Kunlong Xiong, Feng Qiu, Chao Cao

**Affiliations:** Key Laboratory of Respiratory Disease of Ningbo, Department of Respiratory and Critical Care Medicine, The First Affiliated Hospital of Ningbo University, Zhejiang, China

**Keywords:** cadmium, sleep-related disorder, obstructive sleep apnea, daytime sleepiness, trouble sleeping, sleep duration, NHANES

## Abstract

**Background:**

Cadmium is a heavy metal that accumulates in the body due to environmental and occupational exposure. The neurotoxicity of cadmium received increasingly attention in recent years. Sleeping is regulated and coordinated by nervous system, however, little is known about the relationship between cadmium and sleep. This study aimed to examine the relationship between blood cadmium concentrations and sleep-related disorders in US adults.

**Methods:**

This cross-sectional study used data on blood cadmium and sleep from the 2005–2008 and 2015–2020 National Health and Nutrition Examination Survey (NHANES). Weighted multiple regression, generalized weighted modeling, and weighted restricted cubic splines (RCS) were utilized to investigate the association between blood cadmium and sleep outcomes (sleep duration, trouble sleeping, symptoms of obstructive sleep apnea (OSA) and daytime sleepiness). Furthermore, subgroup analyses were conducted to investigate any differences in the associations between age, gender, ethnicity, education level, marital status, smoking status, alcohol consumption, diabetes mellitus (DM), cardiovascular disease (CVD) and hypertension groups.

**Results:**

In 19,152 participants, the median blood cadmium concentration was 0.48 (IQR: 0.28, 0.82)μg/L. Compared with the lowest reference quartile, participants in the higher quartile had a significantly higher risk of insufficient sleeping (<7 h/night) in crude model (OR 1.53, 95% CI 1.33–1.74), Model 1 (OR 1.57, 95% CI 1.38–1.80) and Model 2 (OR 1.45, 95% CI 1.27–1.65). In the unadjusted model, individuals in the highest quartile of cadmium level had a significantly increased risk of OSA symptoms of 53% (OR = 1.53, 95% CI: 1.42, 1.65) compared with participants in the bottom quartile, and this risk increased by 35% (OR = 1.35, 95% CI: 1.23, 1.48) after adjusting for all covariates. Individuals in the highest quartile of cadmium level were 76% more likely to have a trouble sleeping than individuals in the lowest quartile in the unadjusted model (OR = 1.76, 95% CI: 1.31, 1.93), whereas in the fully adjusted model, this likelihood was 86% higher (OR = 1.86, 95% CI: 1.51, 1.96). A similar positive correlation was also observed for cadmium level and daytime sleepiness. However, no relationship was noted between cadmium and excessive sleep duration (≥9 h). A linear dose–response relationship was found between cadmium concentration and the risk of insufficient sleeping (P non-linearity = 0.321), OSA symptoms (P non-linearity = 0.176), trouble sleeping (P non-linearity = 0.682) and daytime sleepiness (P non-linearity = 0.565). Additionally, no significant interactions between cadmium concentrations and subgroup variables were identified (P for interaction>0.05).

**Conclusion:**

Insufficient sleep, symptoms of OSA, trouble sleeping and daytime sleepiness were found to have a positive association with the blood cadmium concentration in US adults. However, further prospective studies are necessary to establish whether there is a causal relationship between these factors.

## Introduction

Sleep is an indispensable physiological function, and everyone spends one-third of their lifetime asleep. With the increasing pace of life and social pressure, an increasing number of people are afflicted with sleep disorders. For example, less than half of Americans sleep as long as recommended by the National Sleep Foundation (1), and sleep disorders affect approximately one-third of the adult population (2). Poor sleep quality has been a risk factor to the development of several chronic conditions including diabetes, cardiovascular disease, obesity and depression (3, 4). Generally, factors related to sleep quality include demographics, socioeconomic status, and lifestyle behaviors, such as alcohol consumption and smoking (5–7). Mounting evidence suggests that the exposure of environmental pollutants may be associated with poor sleep quality and sleep deprivation (5, 6) because absorption of environmental pollutants may induce neurotoxicity via neuroinflammation and oxidative stress (8, 9). Among environmental factors, the roles of heavy metals such as lead, cadmium, and manganese are particularly of interest, given widespread population exposure and their notable neurotoxic effects even at low levels of exposure encountered in the general population.

Cadmium exploitation is a serious global environmental problem that can lead to heavy health and socio-economic burdens. Cadmium is commonly found in household waste, cadmium-containing substances emitted by industry, and even in atmosphere, soil and water (10). The body may absorb cadmium by means of food, smoking, occupational exposure, and other sources (10). The effects of diet and smoking on cadmium levels are significant. Food leafy and root vegetables, cereals and offal are important sources of cadmium and long-term consumption of cereals and root vegetables will increase the accumulation of cadmium within the body through the gastrointestinal tract (11). In addition, as an important component of cigarette smoke, the process of cadmium accumulation is inseparable from the involvement of smoking and cadmium can be inhaled into the lungs through cigarette smoking. It has been established that cadmium levels in smokers are significantly higher than in non-smokers (12), and blood cadmium levels in non-smokers are less than 1 mg/L in most countries, whereas heavy smokers may have blood cadmium levels up to 7-fold higher. There are also differences in blood cadmium levels between different countries, with the average blood cadmium level of adults in the United States being lower than the average blood cadmium level of adults in Italy, Germany, Canada, and Australia (12). The symptoms of poisoning depend on the level of cadmium in the blood and may result in acute and chronic intoxication. It has been determined that cadmium is a type I carcinogen and acts as a proinflammatory cytokine inducer, leading to a chronic inflammatory response (9). Numerous prior investigations have shown an association between exposure to environmental cadmium and a number of illnesses, including osteoporosis (13), renal failure (14), lung fibrosis (15), chronic obstructive pulmonary disease (16), coronary artery disease (17), and respiratory infections (18). However, cadmium toxicity on the function and progression of multiple neurological diseases (such as sleep disorders, stroke, Alzheimer’s disease) is emerging topics and has not been fully examined.

Previous research suggests that cadmium can affect sleep architecture and is a risk factor for the development of obstructive sleep apnea but not a risk factor for sleep bruxism (19). A recent population-based study indicated an association between blood cadmium exposure and trouble sleeping (20). Their analysis of the National Health and Nutrition Examination Survey (NHANES) 2007–2010 waves identified that higher blood cadmium exposure was positively associated with trouble sleeping after adjustment for confounding factors (20). To the best of our knowledge, this is the only large population-based study to investigate cadmium in relation to sleep outcomes at population-level exposures. This present cross-sectional study aimed to extend the work (20) in two distinct and important ways: (i) we will replicate the analyses using more recently collected data in NHANES 2005–2008 and 2015–2020 waves; (ii) we will utilize 3 new measures of sleep health (sleep duration, obstructive sleep apnea (OSA) symptoms, day-time sleepiness) and new questions that are available in the more recent NHANES data.

## Materials and methods

### Study population

We used data from the 2005–2008 and 2015–2020 National Health and Nutrition Examination Survey (NHANES). The NHANES is an ongoing program of study designed by the Centers for Disease Control and Prevention (CDC) for estimating the health and nutritional status of non-institutionalized US civilians. This survey adopts a complex, stratified, and multistage probability sampling design to recruit a nationally representative sample of about 5,000 participants each year. The interview of NHANES covered demographic, socioeconomic, physiological and biochemical indexes as well as other health related issues. Sample information and processing methods from NHANES for epidemiological and health related research can be publicly achieved from the online website.^1^ The National Center for Health Statistics of the US Centers for Disease Control and Prevention authorized the research protocols, and each participant signed written informed consent (21).

**Figure 1:**
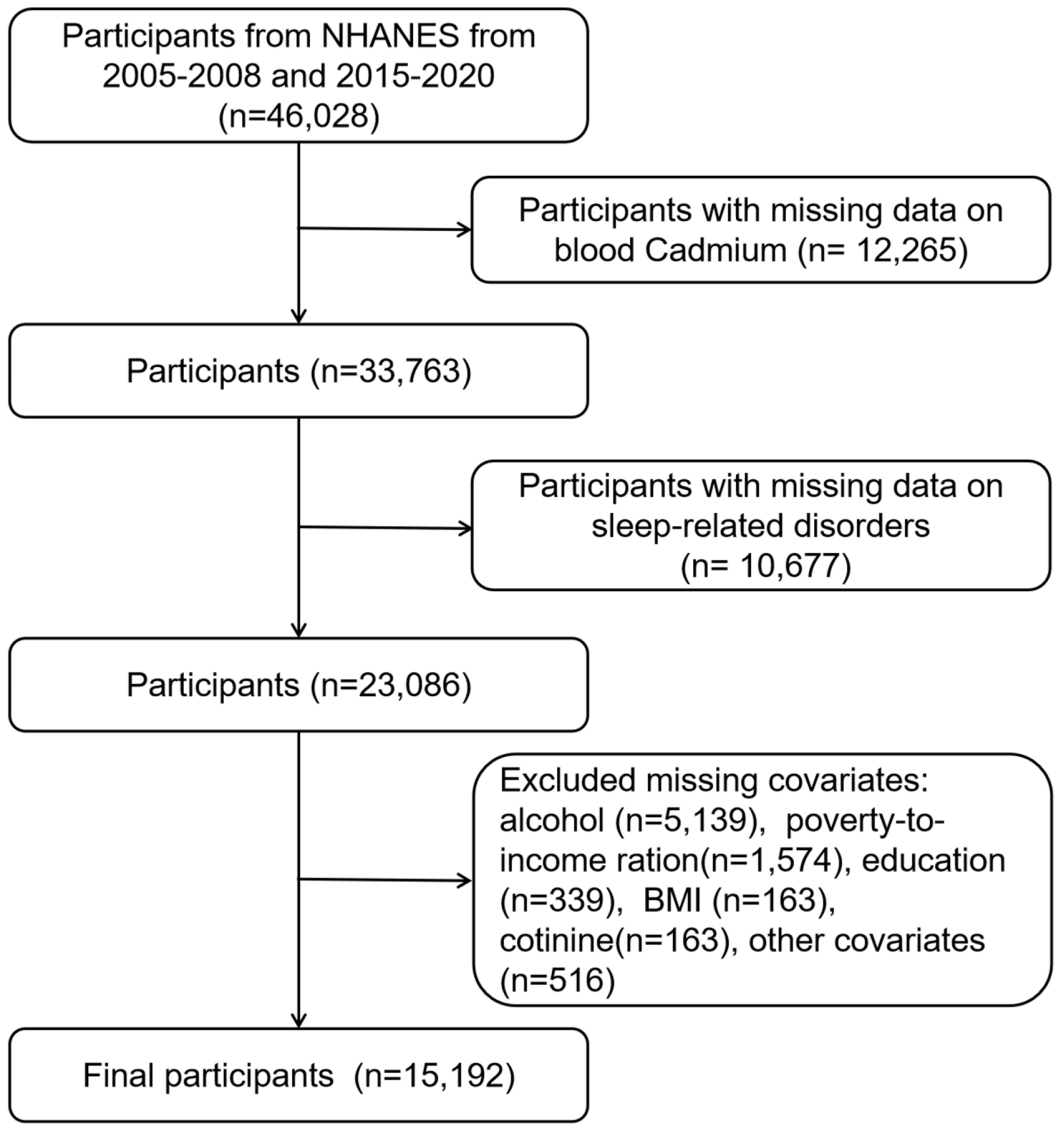
Flowchart for selecting eligible participants in this study.

Figure 1 illustrates the participant selection flowchart. We retrieved information for a total of 46,028 participants from the NHANES datasets of the following years: 2005–2006 (*N* = 10,348), 2007–2008 (*N* = 10,149), 2015–2016 (*N* = 9,971), and 2017–2020 (*N* = 15,560). Due to the coronavirus disease 2019 (COVID-19), the data collection in the 2019–2020 cycle was suspended in March 2020. The 2019-March 2020 pre-pandemic data were combined with the 2017–2018 survey cycle to form nationally representative data. Initially, we excluded participants with missing data on blood cadmium (*n* = 12,265). Subsequently, removed participants with missing data on sleep-related disorders (*n* = 10,677), and participants missing data on covariates (*n* = 7,894). The final study population comprised 15,192 individuals. The participants’ weighted mean age was 47.04 ± 0.35 years, of whom 49.70% (*n* = 7,555) were females and the majority identified as non-Hispanic white (70.32%).

### Definition of the sleep-related disorders

We investigated the following self-reported prevalent outcomes that are related to sleep disorders: sleep duration, obstructive sleep apnea (OSA) symptoms, day-time sleepiness, trouble sleeping. These outcomes were defined in the following ways:

Sleep duration: categorized as insufficient sleep (<7 h/night), normal sleep (7–9 h/night) or excessive sleep (>9 h/night) (22).

OSA symptoms: characterized according to Healthy People (23) and were defined based on 3 questions. These included (1) snoring 3 or more nights a week, (2) experiencing snorting, gasping, or stopping breathing 3 or more nights a week; and (3) feeling excessively sleepy during the day 16 to 30 times a month, even though 7 h of sleep were attained on weekdays or work nights. Participants were determined to have symptoms of OSA if they had any of these symptoms.

Day-time sleepiness: defined according to self-report and was considered to be present if the response frequency was more than or equal to 5 times per month when answering the following questions: “How often did you feel excessively sleepy/feel unrested during the day, no matter how much sleep you get?” (24).

Trouble sleeping: determined by the question, “(Have you/Has the study participant) ever told a doctor or other health professional that (you have/s/he has) trouble sleeping?” The responses to this question were yes/no (25).

### Cadmium assessment

Blood metals’ sampling weights were applied to analyze these data properly. In NHANES 2005–2008 and 2015–2020, cadmium was evaluated by the inductively coupled plasma-mass spectrometer (ICP-MS). Blood samples were collected, stored under frozen (−30°C) conditions, and shipped to the Division of Laboratory Sciences, National Center for Environmental Health, and Centers for Disease Control and Prevention for testing. Detailed information on specific laboratory procedures can be accessed via the following link.^2^ The lower limit of detection (LLOD) of blood cadmium ranged from 0.10 to 0.16 μg/L in 2005–2008 to 2015–2020. For values below the lower limit of detection, the detection limit divided by the square root of two was substituted. In order to approximate a normal distribution, blood cadmium concentrations underwent natural logarithm (ln) transformation for further analyses.

### Covariates determination

Comprehensive data regarding baseline demographic characteristics, lifestyle factors, and medical status were obtained through baseline questionnaires. Demographic characteristics encompassed the following variables: age (continuous variable in years), gender (male/female), race/ethnicity (non-Hispanic white, non-Hispanic black, Mexican American and others), education level (less than high school, completed high school, and more than high school), and the family poverty-to-income ratio (PIR) (continuous) which was employed to assess the economic status. Marital statuses were grouped into married/living with partner, widowed/divorced, or never married.

Lifestyle factors included body mass index (BMI) (continuous), serum cotinine (log-transformed), self-reported smoking status (never-smoker (smoked less than 100 cigarettes in life), former smoker (smoked more than 100 cigarettes in life and smoke not at all currently), and current smoker (had smoked more than 100 cigarettes in life and currently smoke)) (26), and drinking status which was categorized as never (had <12 drinks in lifetime), former (had ≥12 drinks in 1 year and did not drink last year, or did not drink last year but drank ≥12 drinks in lifetime), current heavier drinker (≥3 drinks per day for females, ≥4 drinks per day for males, or binge drinking (≥4 drinks on same occasion for females, ≥5 drinks on same occasion for males) on 5 or more days per month), or current mild/moderate drinker (≤2 drinks per day for females, ≤3 drinks per day for males, or binge drinking ≤2 days per month) (27, 28).

Medical status variables included diabetes mellitus (DM), cardiovascular disease (CVD), hypertension, and general health status. DM was defined as a diagnosis made by a physician or other healthcare professional, glycated hemoglobin (%) greater than 6.5, random blood glucose (mmol/L) equal to or greater than 11.1, two-hour OGTT blood glucose (mmol/l) equal to or greater than 11.1, or use of diabetes medication or insulin (25). Participants who self-reported physician-diagnosed coronary heart disease, heart attack, congestive heart failure, angina or stroke were classified as having CVD (28). Hypertension was defined as systolic blood pressure of at least 140 mmHg, diastolic blood pressure of at least 90 mmHg, or receiving blood pressure control medication (28). General health condition was coded as a categorical variable based on the open-ended question “Would you say (your/SP’s) health in general is…” with participants reporting excellent, very good, good, fair, or poor condition (29).

### Statistical analysis

The statistical analyses adhered to the guidelines (30) established by the CDC, incorporating sampling strata, clusters, and weights provided by NHANES to ensure the representativeness of the findings for the US population. Continuous variables were reported as mean (standard error, SE) if conformed to a normal distribution, and median (interquartile range, IQR) when non-normally distributed whereas categorical variables were expressed as numbers (percentages). Group differences were evaluated through weighted analysis of variance (ANOVA) for continuous variables and the chi-square test for categorical variables. Weighted quartiles of blood cadmium levels were measured according to the distribution within the study population. We also repeated all analyses treating blood cadmium levels as a log-transformed continuous variable. Further, survey-weighted logistic regression model was performed to estimate odds ratios (ORs) and 95% CI for associations between cadmium exposure and the sleep-related disorders.

For the weighted logistic regression, the crude model was adjusted for no covariates. Model 1 was only adjusted for age, gender, and race. Model 2 included additional adjustments for education level, marital status, and PIR. Model 3 further incorporated adjustments for serum cotinine, BMI, smoking status, drinking status, CVD, DM, hypertension, and general health status. In addition, we performed subgroup analyses to determine the association between blood cadmium and sleep problems across age, gender, ethnicity, education level, marital status, smoking status, alcohol consumption, CVD, DM and hypertension groups. Weighted restricted cubic splines (RCS) with 3 knots at the 25th, 50th, and 75th percentiles were used to explore the nonlinear relationships of cadmium exposure and sleep-related disorders in the fully adjusted model.

To ensure the robustness of our findings, we conducted several sensitivity analyses. Firstly, we also re-ran the regression analyses using different cutoffs for the cadmium quartiles so that values below detection limit were only included in the lowest quartile. Secondly, we re-analyzed age, PIR, and BMI by including them as categorical variables in the models. Lastly, unweighted data was utilized to validate the stabilization of the weighted estimates. R software (version 4.2.2) was used for the statistical analyses, and a significance level of a two-sided *p*-value of 0.05 was used to determine the results.

## Results

### Basic characteristics of the study participants

Patients were stratified into quartiles based on weighted blood cadmium levels. The median blood cadmium concentration was 0.48 (IQR: 0.28, 0.82)μg/L, with quartile-specific medians as follows: 0.10 (IQR: 0.07, 0.20) μg/L (quartile 1), 0.26 (IQR: 0.23, 0.30) μg/L (quartile 2), 0.42 (IQR: 0.38, 0.55) μg/L (quartile 3), and 0.96 (IQR: 0.84, 2.09) μg/L (quartile 4). Comparisons of quartile 4 versus quartile 1 revealed that participants with higher cadmium exposures tended to be older, female, lower level of BMI and PIR, higher level of cotinine and higher education level (completed high school), non-Hispanic black groups, widowed/divorced, current smokers, and heavy drinkers. Furthermore, these people with higher cadmium level had higher rates of CVD, hypertension, and fair or poor general health. According to the blood cadmium level quartiles, we also observed differences in sleep problems, high risk for insufficient sleep, OSA symptoms, daytime sleepiness and trouble sleeping (Table 1).

**Table 1:** Weighted characteristics of participants in the NHANES (2005–2008 and 2015–2020) by blood cadmium levels.

Variable	Total (*n* = 15,192)	Quartile 1 (*n* = 3,907)	Quartile 2 (*n* = 3,753)	Quartile 3 (*n* = 3,811)	Quartile 4 (*n* = 3,721)	*p* value
Age (years)	47.04 ± 0.35	41.12 ± 0.41	47.55 ± 0.47	52.46 ± 0.51	48.73 ± 0.40	**< 0.0001**
BMI (kg/m^2^)	29.10 ± 0.11	29.82 ± 0.19	29.57 ± 0.18	28.97 ± 0.18	27.77 ± 0.15	**< 0.0001**
Family poverty-to-income ratio	3.15 ± 0.04	3.37 ± 0.04	3.35 ± 0.06	3.15 ± 0.05	2.64 ± 0.05	**< 0.0001**
Cotinine (ng/mL)	60.38 ± 2.54	17.20 ± 2.04	18.33 ± 2.48	33.53 ± 2.11	191.65 ± 4.59	**< 0.0001**
Gender						**< 0.0001**
Female	7,555(49.70)	1,507(38.57)	1911(50.92)	2,251(65.89)	1886(50.69)	
Male	7,637(50.30)	2,400(61.43)	1842(49.08)	1,560(34.11)	1835(49.31)	
Race/ethnicity						**< 0.0001**
Mexican American	2,393(7.88)	709(9.17)	732(9.49)	602(7.73)	350(4.54)	
Non-Hispanic black	3,304(10.12)	773(8.23)	750(9.52)	829(10.79)	952(12.62)	
Non-Hispanic white	6,732(70.32)	1740(72.76)	1,576(68.93)	1,650(68.41)	1766(70.63)	
Others	2,763(11.67)	685(9.84)	695(12.07)	730(13.07)	653(12.21)	
Education level						**< 0.0001**
Completed high school	3,572(24.29)	817(20.95)	786(20.74)	892(25.21)	1,077(31.74)	
Less than high school	3,471(14.71)	696(9.41)	777(12.64)	911(15.48)	1,087(23.22)	
More than high school	8,149(61.00)	2,394(69.64)	2,190(66.62)	2008(59.31)	1,557(45.04)	
Marital status						**< 0.0001**
Married/living with partner	9,242(64.92)	2,463(65.46)	2,427(68.66)	2,350(67.10)	2002(57.78)	
Never married	2,628(17.00)	910(23.53)	599(14.98)	498(12.10)	621(15.66)	
Widowed/divorced	3,322(18.09)	534(11.02)	727(16.36)	963(20.80)	1,098(26.57)	
Smoking status						**< 0.0001**
Former	3,747(25.29)	779(21.34)	1,018(28.86)	1,283(34.61)	667(16.98)	
Never	8,303(54.09)	3,043(76.47)	2,558(66.18)	2026(50.57)	676(14.64)	
Now	3,142(20.63)	85(2.19)	177(4.95)	502(14.82)	2,378(68.37)	
Alcohol consumption						**< 0.0001**
Current heavier drinker	3,153(21.94)	819(21.27)	667(18.37)	617(17.64)	1,050(31.19)	
Current light/moderate drinker	8,032(55.84)	2,244(60.68)	2047(58.76)	2010(56.11)	1731(45.92)	
Former	2039(12.17)	354(7.56)	502(12.14)	591(14.19)	592(16.19)	
Never	1968(10.06)	490(10.49)	537(10.73)	593(12.06)	348(6.70)	
CVD						**< 0.0001**
No	13,541(91.85)	3,642(95.25)	3,406(92.90)	3,318(89.75)	3,175(88.32)	
Yes	1,651(8.15)	265(4.75)	347(7.10)	493(10.25)	546(11.68)	
DM						**0.04**
No	12,388(86.34)	3,225(87.56)	3,020(85.16)	3,065(85.50)	3,078(86.91)	
Yes	2,804(13.66)	682(12.44)	733(14.84)	746(14.50)	643(13.09)	
Hypertension						**< 0.0001**
No	9,075(64.78)	2,617(71.14)	2,266(65.45)	2,113(59.88)	2079(60.67)	
Yes	6,117(35.22)	1,290(28.86)	1,487(34.55)	1,698(40.12)	1,642(39.33)	
General health						**< 0.0001**
Excellent	2019(16.20)	558(16.98)	555(18.95)	552(17.25)	354(11.00)	
Very good	4,223(32.82)	1,211(36.53)	1,078(34.49)	1,045(31.85)	889(27.05)	
Good	5,489(34.85)	1,397(33.88)	1,349(32.79)	1,359(34.57)	1,384(38.73)	
Fair	2,851(13.46)	634(11.02)	643(11.75)	722(13.91)	852(18.13)	
Poor	610(2.67)	107(1.58)	128(2.02)	133(2.43)	242(5.09)	
Sleep duration						**< 0.0001**
Excessive sleep	966(4.86)	240(4.85)	207(4.06)	248(4.81)	271(5.83)	
Insufficient sleep	4,945(30.81)	1,194(28.78)	1,191(28.75)	1,191(29.13)	1,369(37.50)	
Normal sleep	9,281(64.33)	2,473(66.37)	2,355(67.19)	2,372(66.06)	2081(56.67)	
OSA symptoms						**0.01**
No	10,629(69.82)	2,901(64.26)	2,596(69.18)	2,735(72.03)	2,397(64.42)	
Yes	4,563(30.18)	1,006(25.74)	1,157(30.82)	1,076(27.97)	1,324(35.58)	
Daytime sleepiness						**0.02**
No	11,984(77.91)	3,153(80.70)	2,977(79.01)	3,058(78.75)	2,796(75.14)	
Yes	3,208(22.09)	754(19.30)	776(20.99)	753(21.25)	925(24.86)	
Trouble sleeping						**< 0.001**
No	11,301(72.87)	2,950(74.31)	2,856(75.11)	2,855(71.63)	2,640(69.73)	
Yes	3,891(27.13)	957(25.69)	897(24.89)	956(28.37)	1,081(30.27)	

Quartile 1: < 0.20 μg/L, Quartile 2: 0.21–0.35 μg/L, Quartile 3: 0.36–0.65 μg/L, Quartile 4: > 0.65 μg/L. NHANES, National Health and Nutrition Examination Survey, BMI: body mass index, DM: diabetes mellitus, CVD: cardiovascular disease, OSA: obstructive sleep apnea. Bold values mean significant differences.

### Association between blood cadmium level and sleep duration

Table 2 presents the relationships between the sleep duration classification and blood cadmium level. In the categorical model, compared with the lowest reference quartile, participants in the higher quartile had a significantly higher risk of insufficient sleeping in crude model (OR 1.53, 95% CI 1.33–1.74), Model 1 (OR 1.57, 95% CI 1.38–1.80) and Model 2 (OR 1.45, 95% CI 1.27–1.65). However, the association was insignificant in the most adjusted categorical model (Model 3). In the continuous model, log-transformed cadmium was significantly associated with an increased risk of insufficient sleeping in the fully adjusted model (OR 1.28, 95% CI 1.09–1.68, *p* = 0.02). On the contrary, there was no association between cadmium and excessive sleep duration (≥9 h) compared with normal sleep duration (7–9 h), regardless of whether cadmium is analyzed in quartiles or as a log-transformed continuous variable.

**Table 2:** Weighted logistic regression (OR and 95% CI) of sleep duration with blood cadmium in NHANES (2005–2008 and 2015–2020).

Cadmium	Sleep duration							
	Crude model		Model 1		Model 2		Model 3	
	OR (95%CI)	P	95%CI	P	OR (95%CI)	P	OR (95%CI)	P
Normal sleep *vs* Insufficient sleep								
Quartile 1	ref		ref		ref		ref	
Quartile 2	0.99(0.85,1.14)	0.85	1.03(0.89,1.19)	0.70	1.02(0.89,1.18)	0.74	1.00(0.87,1.16)	0.98
Quartile 3	1.02(0.89,1.16)	0.80	1.09(0.95,1.25)	0.20	1.07(0.93,1.22)	0.33	0.98(0.85,1.14)	0.80
Quartile 4	1.53(1.33,1.74)	**<0.0001**	1.57(1.38,1.80)	**<0.0001**	1.45(1.27,1.65)	**<0.0001**	1.05(0.89,1.25)	0.55
p for trend		**<0.0001**		**<0.0001**		**<0.0001**		0.74
LN cadmium	1.44(1.30,1.60)	**<0.0001**	1.50(1.35,1.67)	**<0.0001**	1.39(1.25,1.55)	**<0.0001**	1.28(1.09,1.68)	**0.02**
Normal sleep *vs* Excessive sleep								
Quartile 1	ref		ref		ref		ref	
Quartile 2	0.83(0.64,1.07)	0.15	0.74(0.58,1.26)	0.20	0.74(0.57,1.45)	0.22	0.74(0.57,0.97)	**0.03**
Quartile 3	1.00(0.78,1.27)	0.97	0.82(0.64,1.05)	0.11	0.76(0.59,1.97)	0.33	0.75(0.57,1.88)	0.34
Quartile 4	1.41(1.04,1.91)	**0.03**	1.21(0.90,1.62)	0.20	0.90(0.67,1.22)	0.50	0.78(0.52,1.16)	0.21
p for trend		**0.03**		0.20		0.56		0.14
LN cadmium	1.52(0.71,2.06)	0.10	1.34(0.98,1.82)	0.06	1.02(0.76,1.37)	0.91	0.88(0.61,1.27)	0.49

Crudel model adjust for: none. Model 1 adjust for: age, gender, race. Model 2 adjust for: model 1 plus education level, marital status and family poverty-to-income ratio. Model 3 adjust for: model 2 plus cotinine level, BMI, smoking status, alcohol consumption, CVD, DM, Hypertension and general health. LN cadmium: Natural log-transformed urinary cadmium; NHANES: The National Health and Nutrition Examination Survey. Bold values mean significant differences.

### Association between blood cadmium level and other sleep-related disorders

The results of the multiple regression analysis were shown in Table 3, including the association of blood cadmium level with OSA symptoms, trouble sleeping and daytime sleepiness.

**Table 3:** Weighted logistic regression (OR and 95% CI) of sleep-related disorders with blood cadmium in NHANES (2005–2008 and 2015–2020).

	Crude model		Model 1		Model 2		Model 3	
Cadmium	95%CI	*P*	95%CI	*P*	95%CI	*P*	95%CI	*P*
Obstructive sleep apnea symptoms								
Quartile 1	ref		ref		ref		ref	
Quartile 2	1.03(0.90,1.18)	0.64	1.05(0.92,1.20)	0.44	1.04(0.91,1.19)	0.57	1.06(0.91,1.23)	0.46
Quartile 3	0.90(0.79,1.02)	0.09	0.92(0.80,1.07)	0.28	0.90(0.79,1.04)	0.16	0.89(0.75,1.06)	0.19
Quartile 4	1.53 (1.42, 1.65)	**<0.001**	1.09(0.95,1.24)	0.21	1.06(0.93,1.21)	0.35	1.35 (1.23, 1.48)	**<0.001**
p for trend		**<0.001**		**<0.001**		**<0.001**		**0.01**
LN cadmium	1.18(1.04,1.27)	**0.02**	1.11 (1.09, 1.13)	**<0.001**	1.10 (1.08, 1.12)	**<0.001**	1.08 (1.05, 1.10)	**0.01**
Trouble sleeping								
Quartile 1	ref		ref		ref		ref	
Quartile 2	0.96(0.84,1.09)	0.51	0.83(0.72,0.96)	**0.01**	0.84(0.73,0.97)	**0.02**	0.82(0.71,0.96)	**0.01**
Quartile 3	1.15(1.01,1.30)	**0.04**	0.89(0.77,1.03)	0.13	0.90(0.78,1.05)	0.17	0.84(0.71,1.00)	0.05
Quartile 4	1.76(1.31,1.93)	**<0.001**	1.06(0.92,1.22)	**0.01**	1.06(0.92,1.24)	**<0.001**	1.86(1.51,1.96)	**0.02**
p for trend		**<0.001**		**<0.001**		**<0.001**		**0.03**
LN cadmium	1.31(1.16,1.48)	**<0.001**	1.12(1.08,1.28)	**<0.001**	1.15(1.12, 1.18)	**<0.001**	1.84(1.48,2.03)	**0.01**
Daytime sleepiness								
Quartile 1	ref		ref		ref		ref	
Quartile 2	0.93(0.80,1.07)	0.29	0.95(0.82,1.10)	**0.01**	0.95(0.82,1.10)	0.49	0.95(0.81,1.10)	0.47
Quartile 3	0.94(0.82,1.09)	**0.02**	0.97(0.84,1.13)	0.71	1.05(0.82,1.10)	0.49	0.91(0.78,1.07)	0.26
Quartile 4	1.10(0.96,1.26)	**0.01**	1.12(0.97,1.29)	0.12	1.32(0.88,1.18)	**<0.001**	0.80(0.64,1.01)	0.06
p for trend		**<0.001**		**<0.001**		**0.01**		0.08
LN cadmium	1.14(1.04,1.31)	**<0.001**	1.17(1.02,1.35)	**0.03**	1.28(1.13,1.55)	**0.01**	1.02(0.76,1.12)	0.09

Crudel model adjust for: none. Model 1 adjust for: age, gender, race. Model 2 adjust for: model 1 plus education level, marital status and family poverty-to-income ratio. Model 3 adjust for: model 2 plus cotinine level, BMI, smoking status, alcohol consumption, CVD, DM, Hypertension and general health. Bold values mean significant differences.

Our results indicated that higher cadmium level was connected with a higher risk of OSA symptoms. After transforming blood cadmium level into quartiles, this relation maintained its statistical significance. In the unadjusted model, individuals in the highest quartile of cadmium level had a significantly increased risk of OSA symptoms of 53% (OR = 1.53, 95% CI: 1.42, 1.65; p for trend <0.001) compared with participants in the bottom quartile of cadmium level, and this risk increased by 35% (OR = 1.35, 95% CI: 1.23, 1.48; p for trend <0.001) after adjusting for all covariates. When considering serum cadmium as a continuous variable, this relation was still significant in all models (crude model, OR = 1.18, 95% CI: 1.04, 1.27, *p* = 0.02, Model 1, OR = 1.11, 95% CI: 1.09, 1.13, *p* < 0.001; Model 2, OR = 1.10, 95% CI: 1.08, 1.12, *p* < 0.001; Model 3, OR = 1.08, 95% CI: 1.05, 1.10, *p* = 0.01).

A similar positive correlation was also observed for cadmium level with trouble sleeping. Individuals in the highest quartile of cadmium level were 76% more likely to have a trouble sleeping than individuals in the lowest quartile in the unadjusted model (OR = 1.76, 95% CI: 1.31, 1.93; p for trend <0.001), whereas in the fully adjusted model, this likelihood was 86% higher (OR = 1.86, 95% CI: 1.51, 1.96; p for trend = 0.03). This positive association persisted when cadmium level was transformed into continuous variable, too. We noted a significant correlation between cadmium level and trouble sleeping in all models (Crude model, OR = 1.31, 95% CI: 1.16, 1.48, *p* < 0.001, Model 1, OR = 1.12, 95% CI: 1.08, 1.28, p < 0.001; Model 2, OR = 1.15, 95% CI: 1.12, 1.18, *p* < 0.001; Model 3, OR = 1.84, 95% CI: 1.48, 2.03, *p* = 0.01).

In the continuous model, our results showed a positive correlation between cadmium level and daytime sleepiness in all models (Crude model, OR = 1.14, 95% CI: 1.04, 1.31, p < 0.001, Model 1, OR = 1.17, 95% CI: 1.02, 1.35, *p* = 0.03; Model 2, OR = 1.28, 95% CI: 1.13, 1.55, *p* = 0.01), however, this correlation was not significant in the fully adjusted model (Table 3). This correlation remained significant even when cadmium was converted to a quartile variable. However, the relation of cadmium level to daytime sleepiness was not statistically significant after adjusting for all covariates, regardless of whether cadmium was a continuous variable or a categorical variable.

### Non-linear analysis of the association of serum cadmium level and sleep-related disorders

**Figure 2:**
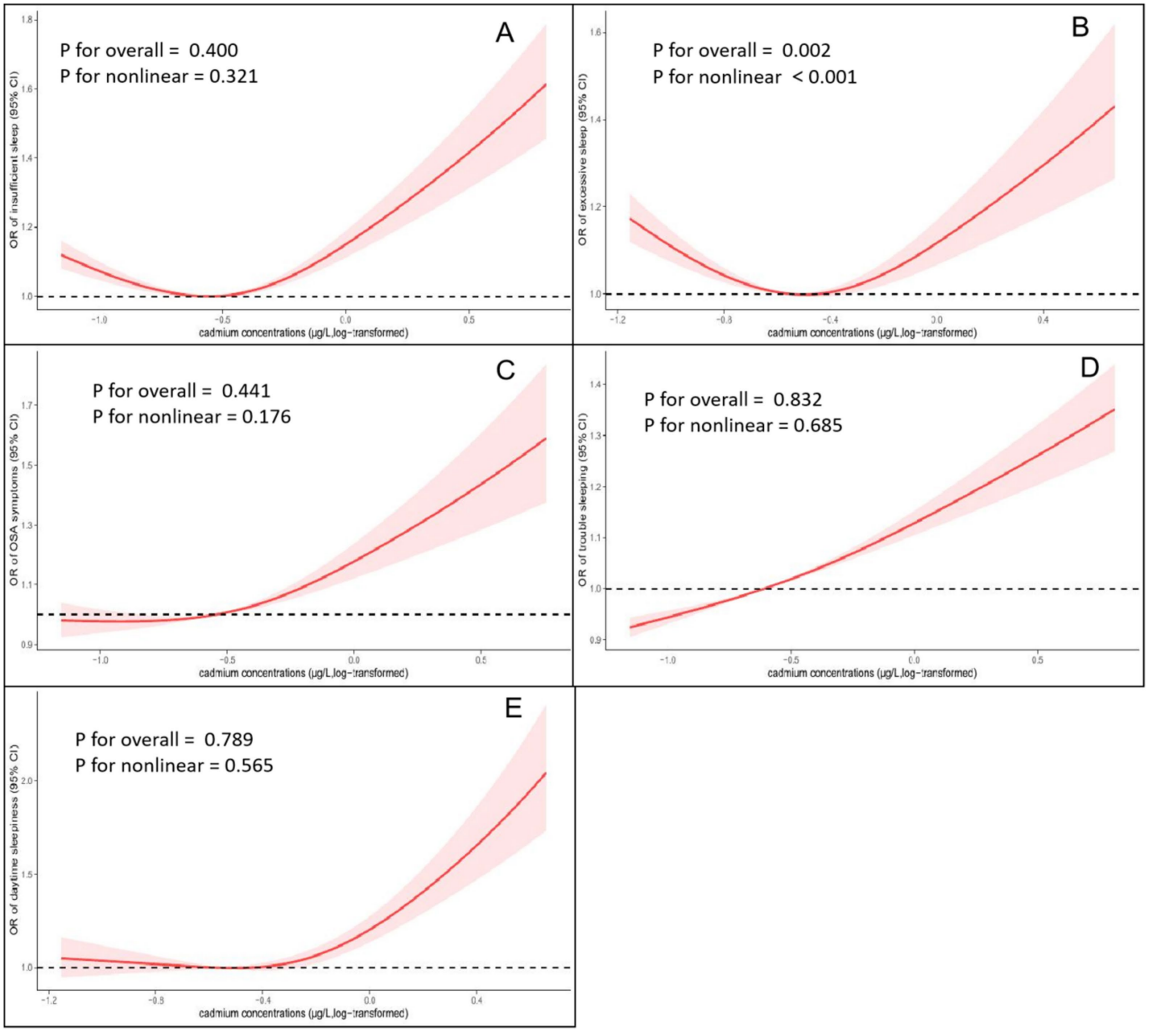
Non-linear relationship between blood cadmium concentrations and the prevalence of sleep-related disorders by restricted cubic spline fitting (RCS). (A) Cadmium concentrations and insufficient sleep. (B) Cadmium concentrations and excessive sleep. (C) Cadmium concentrations and obstructive sleep apnea (OSA) symptoms. (D) Cadmium concentrations and trouble sleeping; (E) Cadmium concentrations and daytime sleepiness. Analyses were adjusted for covariates age, gender, race, education level, marital status, family poverty-to-income ratio, cotinine level, BMI, smoking status, alcohol consumption, CVD, DM, Hypertension and general health.

To explore the non-linear exposure-response relationship between cadmium exposure and sleep disturbances, we analysed this relationship using RCS regression. The results of multivariate linear regression with RCS are presented in Figure 2. Except for excessive sleep, significant linear association was found between log-transformed cadmium concentrations and the risk of insufficient sleeping (P non-linearity = 0.321), OSA symptoms (P non-linearity = 0.176), trouble sleeping (P non-linearity = 0.685) and daytime sleepiness (P non-linearity = 0.565). According to the results of the RCS regression models, higher blood concentration of cadmium was associated with a higher risk of sleep-related disorders, particularly when the concentration was above the mean.

### Subgroup and interaction analyses

The subgroup analyses were conducted to determine whether the association between cadmium exposure and the risk of insufficient sleeping was consistent in different groups (Table 4). After adjusting for the comprehensive model, when compared to the group with the lowest quartile cadmium concentrations, we observed that the group with the highest quartile serum cadmium level was positively correlated with risk of insufficient sleeping in participants aged between 40 and 59 years old (OR 2.11, 95% CI 2.05–2.17, *p* < 0.001), males (OR 3.14, 95% CI 2.11–6.50, *p* < 0.001), individuals of Non-Hispanic white (OR 3.78, 95% CI 2.07–5.08, p < 0.001) and other racial backgrounds (OR 3.63, 95% CI 2.27–6.18, *p* = 0.01), had an education level of more than high school (OR 2.69 95% CI 1.38–5.25, *p* = 0.01), current smokers (OR 3.56, 95% CI 1.84–6.31, *p* = 0.01), current heavy drinkers (OR 5.61 95% CI 2.77–11.40, *p* < 0.001) and those with hypertension (OR 2.08 95% CI 2.02–3.14, *p* = 0.04). On the contrary, there were no significant associations in subgroup analyses stratified by marital status, CVD and DM. Additionally, no significant interactions between cadmium concentrations and subgroup variables were identified (P for interaction>0.05). Similar results were observed between cadmium exposure and the risk of other sleep-related disorders (Supplementary Tables S1–S3), which indicated that the main analysis results can be considered stable.

**Table 4:** Subgroup analysis for the association between blood cadmium level and the risk of insufficient sleep.

Subgroups	Quartile 1	Quartile 2	Quartile 3	Quartile 4	P–t	P-int
Age (years)						0.17
20–39	ref	1.11 (0.84–1.47)	1.34 (1.04–1.73)	1.14(1.08,2.21)	0.08	
40–59	ref	1.00 (0.75–1.33)	1.33 (1.05–1.70)	2.11(2.05,2.17)	**<0.001**	
≥60	ref	1.43 (1.00–2.04)	1.28 (0.86–1.90)	1.05(1.01,1.11)	0.30	
Gender						0.41
Female	ref	1.20 (0.46, 1.78)	1.47 (0.92, 2.38)	1.35 (0.56, 2.59)	0.20	
Male	ref	1.28 (0.53, 1.85)	2.53 (2.29, 4.16)	3.14 (2.11, 6.50)	**<0.001**	
Race/ethnicity						0.90
Mexican American	ref	0.05(0.01,0.11)	0.06(0.01,0.12)	0.04(0.03,0.12)	0.09	
Non-Hispanic black	ref	0.04(0.02,0.09)	0.08(0.02,0.15)	0.04(0.03,0.12)	0.38	
Non-Hispanic white	ref	1.06 (0.77, 1.82)	2.44 (1.36, 4.73)	3.78 (2.07, 5.08)	**<0.001**	
Others	ref	1.04 (0.54, 2.41)	1.66 (0.78, 2.49)	3.63 (2.27, 6.18)	**0.01**	
Education level						0.57
More than high school	ref	1.68 (0.95, 3.00)	1.83 (1.00, 3.35)	2.69 (1.38, 5.25)	**0.01**	
Completed high school	ref	0.05(0.01,0.14)	0.04(0.03,0.05)	0.08(0.03,0.14)	0.30	
Less than high school	ref	0.87 (0.61, 1.56)	2.26 (1.39, 5.54)	1.07 (1.32, 3.66)	0.11	
Marital status						0.33
Married/living with partner	ref	1.32 (0.40, 4.34)	2.40 (0.83, 6.91)	1.91 (0.60, 6.15)	0.31	
Never married	ref	0.72 (0.43 2.34)	1.40 (0.78, 4.54)	1.94 (1.60, 3.51)	0.24	
Widowed/divorced	ref	1.57 (0.89, 2.77)	2.04 (1.12, 3.73)	1.52 (1.46, 3.31)	0.06	
Smoking status						0.54
Never	ref	1.66 (0.86, 3.14)	2.49 (1.36, 6.45)	1.45 (0.60, 3.53)	0.07	
Former	ref	0.12(0.10,0.45)	0.23(0.10,0.45)	0.65(0.28,0.99)	0.35	
Now	ref	1.32 (0.67, 2.47)	1.78 (0.93, 2.86)	3.56 (1.84, 6.31)	**0.01**	
Alcohol consumption						0.81
Current heavier drinker	ref	1.81 (0.99, 3.32)	3.10 (1.67, 5.73)	5.61 (2.77, 11.40)	**<0.001**	
Current light/moderate drinker	ref	1.34 (0.69, 2.43)	2.42 (1.47, 4.36)	2.52 (1.58, 6.74)	0.06	
Former	ref	0.77(0.44,1.31)	1.35(0.67,2.48)	0.92(0.34,2.56)	0.09	
Never	ref	0.67(0.50,2.11)	0.53(0.17,2.13)	0.86(0.45,1.16)	0.28	
CVD						0.70
Yes	ref	0.99 (0.71,1.38)	1.01 (0.73,1.41)	0.29(0.12,0.79)	0.16	
No	ref	1.10 (0.86,1.41)	1.39 (1.12,1.73)	1.09 (0.42,2.34)	0.23	
DM						0.13
Yes	ref	0.95 (0.75,1.19)	1.31 (1.02,1.68)	1.03(0.46,3.12)	0.08	
No	ref	1.28 (0.91,1.80)	1.25 (0.94,1.66)	1.12(1.08,1.16)	0.11	
Hypertension						0.10
Yes	ref	1.06 (0.84,1.33)	1.47 (1.15,1.89)	2.08(2.02,3.14)	**0.04**	
No	ref	1.12 (0.83–1.51)	1.09 (0.80–1.49)	1.11(1.07,1.16)	0.57	

Quartile 1: < 0.20 μg/L, Quartile 2: 0.21–0.35 μg/L, Quartile 3: 0.36–0.65 μg/L, Quartile 4: > 0.65 μg/L. DM: diabetes mellitus, CVD: cardiovascular disease, p–t, p for trend; p-int, p for interaction. Bold values mean significant differences.

### Sensitivity analyses

Results did not meaningfully change when using different quartile cutoffs for blood cadmium. Similar results were observed when modeling age, PIR, and BMI as categorical rather than continuous variables (Supplementary Tables S4, S5). Repetition of analyses using unweighted data was held concordant and did not deviate significantly from the initial results (Supplementary Table S6).

## Discussion

This study examined the relationship between cadmium exposure and sleep problems in a representative sample of adults from the US population using a cross-sectional approach. The results revealed a dose–response relationship, demonstrating that elevated levels of cadmium in the blood were in positive correlation with an increased risk of sleep-related disorders, including insufficient sleeping, OSA symptoms, day-time sleepiness and trouble sleeping in US population. However, blood cadmium concentration was observed to have no association with excessive sleep. Results were consistent regardless of the method of statistical adjustment, standardization or stratified analyses.

Cadmium is ubiquitous in the earth’s crust and is primarily emitted into the surrounding ecosystem via waste emissions from mining and smelting processes (10). Cadmium particles in industrial emissions settle into the atmosphere, soil, and water, leading to elevated regional cadmium levels. Subsequently, cadmium available from the environment would be transferred to crops, where it eventually accumulates within the body through the gastrointestinal tract or is inhaled into the lungs through respiration (10). Diet is the principal cause of public contamination with cadmium, while smoking directly introduces cadmium into the body (10). Cadmium accumulation in the body could cause adverse health consequences. The kidneys are the major site of cadmium accumulation in the body. This in turn leads to nephrotoxicity and renal tubular damage, causing numerous negative health outcomes (14, 31). Furthermore, chronic cadmium toxicity is associated with clinical manifestations such as coronary diseases, osteomalacia, anemia and immune-toxic effects (17, 32, 33). Cadmium has also been found to be neurotoxic. The recent literature states that cadmium has a strictly dose-dependent effect on neurons: Cadmium can gradually cause cell injury, cell death and organ failure at high doses. Converging evidence indicates that exposure to cadmium can cause sleep disorders by cadmium-dependent neurotoxicity. Utilizing data from 35,108 Korean adults, Yeo et al. (34) reported a positive association between serum cadmium and short sleep (sleep less than 6 h per night), which was in agreement with the outcome of the present study. Past studies evaluating the serum levels of heavy metals in patients with obstructive sleep apnea (OSA) revealed that, among others, the serum level of cadmium was increased in the study group (compared to controls), possibly due to oxidative stress and inflammation (35). A cohort study in rural China reported that long-term exposure to cadmium was associated with an increased risk of poor sleep quality and higher global Pittsburgh Sleep Quality Index (PSQI) scores in approximately 27,000 adults (6). Recently, Kim et al. (36) also suggested that inhalation of cadmium in particulate matter may adversely affect sleep quality. Our results are consistent with previous investigations.

The association between cadmium exposure and sleep disorders may be explained by heavy metal neurotoxicity. One animal study explored the effect of cadmium exposure on brain structure and function in rats, results showed that one single injections of cadmium chloride lead to significant changes of wakefulness-sleep cycle (37). Neurodegeneration due to the generation of reactive oxygen species (ROS) by other heavy metals, whether directly or not has been previously reported (38, 39). From the mechanism, an increase and accumulation of ROS after cadmium exposure might help to explain this phenomenon. Another rats study verified this hypothesis that cadmium-induced sleep disturbance as a consequence of oxidative stress (40). Considering that oxidized glutathione is one of endogenous sleep substances, cadmium could induce abnormal sleep wake cycle by occupying intrinsic sleep-inducing neurotransmitters. In detail, the dysregulated activity of neurotransmitters involved in sleep regulation such as dopamine and serotonin (41) could be affected by high doses of metal exposure in the brain. Accounting for the facts that neurotransmitters regulating circadian rhythms also played important roles in neurodegenerative disorders (42), knowing the mechanism of cadmium exposure on sleep might also help to prevent metal induced sleep problems and its related diseases. On the other hand, cadmium may increase inflammation in the tissues of the respiratory tract, favoring the narrowing of the upper airway during sleep and affecting respiratory control stability/loop gain, the respiratory arousal threshold and upper airway muscle function via neurotoxic activity. However, these hypotheses require further research.

Our study adds to the limited literature on the relationship between cadmium and sleep in the US population. We utilized the most recent NHANES dataset, which is nationally representative, making the findings applicable to US adults. In addition, the study adjusted for potential social demographic and lifestyle confounders to obtain more robust results. Furthermore, we use weighted sample analyses to examine the association. On this basis, the results were more generalizable. At the same time, several limitations should be noted when interpreting this article. First, we diagnose sleep-related disorders on the basis of some typical symptoms found in NHANES questionnaire, such as daytime sleepiness, apnea, snoring, etc. And recall or self-report bias becomes a limitation with the use of questionnaires to collect information, which also does not adequately account for the psychological characteristics of the participants, but using the NHANES database, there have been several studies of sleep-related disorders (38). Second, because only Americans were included in the research, findings may not fully apply to other nations or areas, such as Asia and Africa. Third, we cannot establish causality on the basis of this cross-sectional study, and intervention studies are needed for further confirmation.

## Conclusion

This study found a positive association between environmental cadmium exposure levels and an increased risk of sleep-related disorders, including insufficient sleeping, OSA symptoms, day-time sleepiness and trouble sleeping in US population, highlighting the importance of recognizing the harmful effects of cadmium on the sleep quality of adults at the community level. Effective measures should be taken to control cadmium pollution and reduce environmental cadmium exposure to mitigate its health hazards. However, we were unable to establish a causal association between blood cadmium and sleep disorders owing to the cross-sectional research design. Consequently, further research is required to determine the potential consequences of blood cadmium on sleep disorders in the future.

## Data Availability

The original contributions presented in the study are included in the article/Supplementary material, further inquiries can be directed to the corresponding author.

## Footnotes

^1^ https://www.cdc.gov/nchs/nhanes/index.htm

^2^ https://www.cdc.gov/nchs/nhanes/
