# Supplementary material for: Analysis of the relationship between sleep-related disorders and cadmium in the US population
Source: Front Public Health. 2024 Oct 25;12:1476383. doi: 10.3389/fpubh.2024.1476383 (PMC11544537; doi:10.3389/fpubh.2024.1476383)
Supplement: Supplementary file 1 [file Data_Sheet_1.doc]

| **Supplementary Table 1. Subgroup analysis for the association between blood cadmium level and the risk of daytime sleepiness** | | | | | | |
| --- | --- | --- | --- | --- | --- | --- |
| **Subgroups** | **Quartile 1** | **Quartile 2** | **Quartile 3** | **Quartile 4** | **P-t** | **P-int** |
| **Age (years)** |  |  |  |  |  | 0.04 |
| 20–39 | ref | 1.09(0.86,1.38) | 1.16(0.89,1.51) | 1.34(1.12,1.59) | **0.005** |  |
| 40–59 | ref | 0.87(0.67,1.13) | 1.13(0.88,1.45) | 1.24(0.95,1.62) | **0.04** |  |
| ≥60 | ref | 0.80(0.60,1.06) | 0.63(0.49,0.81) | 0.68(0.52,0.88) | 0.11 |  |
| **Gender** |  |  |  |  |  | 0.39 |
| Female | ref | 0.80(0.65,0.98) | 0.79(0.65,0.95) | 0.94(0.76,1.16) | 0.55 |  |
| Male | ref | 0.96(0.78,1.18) | 0.95(0.77,1.17) | 1.15(0.93,1.43) | 0.29 |  |
| **Race/ethnicity** |  |  |  |  |  | 0.25 |
| Mexican American | ref | 0.91(0.66,1.25) | 0.82(0.56,1.21) | 0.96(0.66,1.38) | 0.51 |  |
| Non-Hispanic black | ref | 0.92(0.73,1.17) | 1.00(0.75,1.34) | 1.01(0.78,1.31) | 0.79 |  |
| Non-Hispanic white | ref | 0.93(0.77,1.11) | 0.98(0.82,1.18) | 1.17(0.99,1.39) | 0.10 |  |
| Others | ref | 0.97(0.67,1.43) | 0.75(0.50,1.10) | 0.77(0.54,1.11) | 0.06 |  |
| **Education level** |  |  |  |  |  | 0.29 |
| More than high school | ref | 0.92(0.77,1.10) | 0.90(0.75,1.07) | 1.09(0.91,1.31) | 0.73 |  |
| Completed high school | ref | 1.00(0.74,1.36) | 0.98(0.72,1.32) | 1.00(0.74,1.35) | 0.95 |  |
| Less than high school | ref | 0.89(0.64,1.24) | 1.15(0.82,1.62) | 1.42(1.08,1.86) | <0.001 |  |
| **Marital status** |  |  |  |  |  | 0.85 |
| Married/living with partner | ref | 0.95(0.78,1.17) | 0.91(0.74,1.12) | 1.13(0.91,1.41) | 0.47 |  |
| Never married | ref | 0.84(0.59,1.20) | 1.02(0.69,1.52) | 1.08(0.83,1.41) | 0.53 |  |
| Widowed/divorced | ref | 1.00(0.67,1.50) | 1.05(0.76,1.46) | 1.03(0.74,1.42) | 0.8 |  |
| **Smoking status** |  |  |  |  |  | **0.03** |
| Never | ref | 0.94(0.79,1.13) | 0.90(0.74,1.09) | 0.73(0.55,0.98) | 0.06 |  |
| Former | ref | 0.75(0.57,0.99) | 0.67(0.50,0.89) | 0.72(0.50,1.03) | **0.03** |  |
| Now | ref | 2.13(0.87,5.23) | 2.65(1.18,5.95) | 2.19(1.04,4.62) | 0.39 |  |
| **Alcohol consumption** |  |  |  |  |  | 0.36 |
| Current heavier drinker | ref | 0.91(0.62,1.33) | 0.98(0.65,1.46) | 1.30(1.02,1.66) | **0.03** |  |
| Current light/moderate drinker | ref | 0.92(0.74,1.15) | 0.88(0.71,1.09) | 0.90(0.72,1.13) | 0.28 |  |
| Former | ref | 0.81(0.56,1.15) | 1.03(0.70,1.50) | 1.19(0.85,1.68) | 0.15 |  |
| Never | ref | 1.10(0.70,1.72) | 1.02(0.65,1.60) | 0.80(0.45,1.45) | 0.55 |  |
| CVD |  |  |  |  |  | 0.81 |
| Yes | ref | 0.76(0.50,1.16) | 0.82(0.57,1.19) | 0.89(0.61,1.29) | 0.81 |  |
| No | ref | 0.93(0.80,1.08) | 0.92(0.78,1.09) | 1.08(0.93,1.27) | 0.49 |  |
| DM |  |  |  |  |  | 0.24 |
| Yes | ref | 0.72(0.51,1.01) | 0.69(0.49,0.98) | 0.97(0.68,1.40) | 0.72 |  |
| No | ref | 0.96(0.82,1.13) | 0.99(0.84,1.16) | 1.12(0.97,1.30) | 0.18 |  |
| Hypertension |  |  |  |  |  | 0.07 |
| Yes | ref | 0.87(0.68,1.11) | 0.73(0.56,0.95) | 1.04(0.82,1.32) | 0.96 |  |
| No | ref | 0.94(0.79,1.13) | 1.07(0.90,1.27) | 1.09(0.93,1.26) | 0.19 |  |

Quartile 1: < 0.20 μg/L, Quartile 2: 0.21-0.35μg/L, Quartile 3: 0.36-0.65μg/L, Quartile 4: > 0.65μg/L

DM: diabetes mellitus, CVD: cardiovascular disease, p-t, p for trend; p-int, p for interaction

| **Supplementary Table 2. Subgroup analysis for the association between blood cadmium level and the risk of trouble sleeping** | | | | | | |
| --- | --- | --- | --- | --- | --- | --- |
| **Subgroups** | **Quartile 1** | **Quartile 2** | **Quartile 3** | **Quartile 4** | **P-t** | **P-int** |
| **Age (years)** |  |  |  |  |  | 0.06 |
| 20–39 | ref | 0.89(0.67,1.18) | 0.91(0.71,1.18) | 1.45(1.13,1.86) | **0.02** |  |
| 40–59 | ref | 0.89(0.72,1.10) | 1.05(0.87,1.27) | 1.22(0.99,1.49) | 0.13 |  |
| ≥60 | ref | 0.71(0.53,0.97) | 0.80(0.62,1.04) | 0.66(0.49,0.88) | **0.03** |  |
| **Gender** |  |  |  |  |  | 0.33 |
| Female | ref | 1.01(0.81,1.25) | 1.04(0.86,1.27) | 1.15(0.94,1.41) | 0.18 |  |
| Male | ref | 0.83(0.68,1.00) | 1.02(0.87,1.21) | 1.20(1.01,1.43) | **0.01** |  |
| **Race/ethnicity** |  |  |  |  |  | 0.07 |
| Mexican American | ref | 1.34(0.93,1.95) | 1.57(1.13,2.19) | 1.94(1.30,2.92) | **<0.001** |  |
| Non-Hispanic black | ref | 0.99(0.77,1.26) | 0.95(0.75,1.20) | 1.30(1.05,1.62) | 0.20 |  |
| Non-Hispanic white | ref | 0.96(0.81,1.14) | 1.22(1.03,1.45) | 1.22(1.04,1.43) | 0.30 |  |
| Others | ref | 0.88(0.62,1.26) | 0.80(0.54,1.19) | 1.11(0.81,1.51) | 0.69 |  |
| **Education level** |  |  |  |  |  | 0.01 |
| More than high school | ref | 0.86(0.74,1.01) | 1.03(0.87,1.22) | 1.23(1.02,1.49) | 0.06 |  |
| Completed high school | ref | 1.30(0.93,1.81) | 1.54(1.19,1.98) | 1.33(0.99,1.79) | 0.08 |  |
| Less than high school | ref | 1.27(0.87,1.85) | 1.61(1.16,2.25) | 2.13(1.53,2.97) | **<0.0001** |  |
| **Marital status** |  |  |  |  |  | 0.05 |
| Married/living with partner | ref | 0.83(0.71,0.96) | 1.13(0.97,1.32) | 1.19(1.02,1.39) | 0.06 |  |
| Never married | ref | 1.08(0.75,1.55) | 0.95(0.72,1.25) | 1.14(0.85,1.54) | 0.50 |  |
| Widowed/divorced | ref | 1.18(0.88,1.57) | 1.00(0.73,1.37) | 1.12(0.83,1.52) | 0.72 |  |
| **Smoking status** |  |  |  |  |  | **0.02** |
| Never | ref | 0.87(0.74,1.02) | 1.09(0.94,1.27) | 0.87(0.66,1.16) | 0.96 |  |
| Former | ref | 1.01(0.73,1.38) | 1.12(0.82,1.52) | 0.90(0.66,1.23) | 0.94 |  |
| Now | ref | 1.71(0.72,4.05) | 1.46(0.73,2.93) | 2.20(1.12,4.32) | **0.01** |  |
| **Alcohol consumption** |  |  |  |  |  | 0.03 |
| Current heavier drinker | ref | 1.35(0.95,1.93) | 1.46(1.07,1.98) | 1.69(1.22,2.35) | **0.001** |  |
| Current light/moderate drinker | ref | 0.89(0.77,1.03) | 1.02(0.85,1.22) | 1.02(0.85,1.21) | 0.64 |  |
| Former | ref | 0.82(0.55,1.22) | 1.23(0.83,1.82) | 1.63(1.08,2.44) | 0.31 |  |
| Never | ref | 0.90(0.61,1.33) | 1.27(0.85,1.90) | 1.24(0.77,2.00) | 0.20 |  |
| **CVD** |  |  |  |  |  | 0.26 |
| Yes | ref | 0.90(0.58,1.39) | 0.78(0.51,1.19) | 1.06(0.72,1.56) | 0.72 |  |
| No | ref | 0.94(0.82,1.08) | 1.14(0.99,1.31) | 1.19(1.04,1.37) | 0.44 |  |
| **DM** |  |  |  |  |  | 0.33 |
| Yes | ref | 0.74(0.53,1.05) | 0.92(0.67,1.27) | 1.10(0.79,1.54) | 0.43 |  |
| No | ref | 0.99(0.86,1.15) | 1.18(1.03,1.34) | 1.28(1.11,1.48) | **<0.001** |  |
| **Hypertension** |  |  |  |  |  | 0.70 |
| Yes | ref | 0.92(0.75,1.14) | 1.01(0.83,1.23) | 1.09(0.90,1.32) | 0.29 |  |
| No | ref | 0.91(0.77,1.08) | 1.10(0.91,1.32) | 1.23(1.02,1.49) | **0.22** |  |

Quartile 1: < 0.20 μg/L, Quartile 2: 0.21-0.35μg/L, Quartile 3: 0.36-0.65μg/L, Quartile 4: > 0.65μg/L

DM: diabetes mellitus, CVD: cardiovascular disease, p-t, p for trend; p-int, p for interaction

| **Supplementary Table 3. Subgroup analysis for the association between blood cadmium level and the risk of OSA symptoms** | | | | | | |
| --- | --- | --- | --- | --- | --- | --- |
| **Subgroups** | **Quartile 1** | **Quartile 2** | **Quartile 3** | **Quartile 4** | **P-t** | **P-int** |
| **Age (years)** |  |  |  |  |  | 0.11 |
| 20–39 | ref | 1.01(0.81,1.27) | 0.97(0.77,1.22) | 1.49(1.18,1.87) | **0.01** |  |
| 40–59 | ref | 0.89(0.73,1.09) | 0.80(0.64,1.00) | 0.89(0.72,1.11) | 0.20 |  |
| ≥60 | ref | 0.97(0.71,1.33) | 0.70(0.53,0.92) | 0.69(0.53,0.90) | **<0.001** |  |
| **Gender** |  |  |  |  |  | 0.42 |
| Female | ref | 1.09(0.91,1.32) | 0.92(0.75,1.14) | 1.17(0.97,1.42) | 0.38 |  |
| Male | ref | 1.08(0.92,1.28) | 1.07(0.93,1.24) | 1.10(0.93,1.30) | 0.26 |  |
| **Race/ethnicity** |  |  |  |  |  | 0.56 |
| Mexican American | ref | 1.05(0.82,1.36) | 1.20(0.89,1.63) | 0.98(0.74,1.29) | 0.56 |  |
| Non-Hispanic black | ref | 1.02(0.83,1.27) | 0.97(0.75,1.26) | 1.06(0.84,1.35) | 0.69 |  |
| Non-Hispanic white | ref | 1.03(0.86,1.23) | 0.87(0.73,1.04) | 1.11(0.93,1.31) | 0.67 |  |
| Others | ref | 1.06(0.75,1.49) | 0.84(0.58,1.21) | 0.93(0.67,1.28) | 0.36 |  |
| **Education level** |  |  |  |  |  | 0.64 |
| More than high school | ref | 1.07(0.89,1.28) | 0.87(0.74,1.03) | 1.08(0.92,1.26) | 0.88 |  |
| Completed high school | ref | 0.93(0.70,1.23) | 0.93(0.72,1.22) | 0.93(0.74,1.17) | 0.58 |  |
| Less than high school | ref | 1.05(0.79,1.41) | 0.87(0.63,1.21) | 1.15(0.88,1.50) | 0.44 |  |
| **Marital status** |  |  |  |  |  | 0.004 |
| Married/living with partner | ref | 1.00(0.84,1.18) | 0.89(0.76,1.05) | 1.04(0.89,1.22) | 0.91 |  |
| Never married | ref | 1.02(0.71,1.47) | 0.79(0.56,1.13) | 1.83(1.35,2.49) | **0.002** |  |
| Widowed/divorced | ref | 0.94(0.66,1.35) | 0.74(0.55,1.02) | 0.78(0.56,1.09) | 0.07 |  |
| **Smoking status** |  |  |  |  |  | 0.31 |
| Never | ref | 1.07(0.92,1.25) | 0.82(0.70,0.97) | 0.63(0.50,0.80) | **<0.001** |  |
| Former | ref | 0.82(0.64,1.05) | 0.77(0.61,0.98) | 0.75(0.54,1.03) | **0.03** |  |
| Now | ref | 1.19(0.57,2.47) | 0.88(0.45,1.73) | 0.92(0.49,1.72) | 0.46 |  |
| **Alcohol consumption** |  |  |  |  |  | 0.02 |
| Current heavier drinker | ref | 0.95(0.69,1.30) | 1.01(0.72,1.41) | 1.36(1.07,1.72) | **0.11** |  |
| Current light/moderate drinker | ref | 0.98(0.83,1.15) | 0.81(0.69,0.95) | 0.87(0.75,1.02) | **0.02** |  |
| Former | ref | 1.20(0.74,1.92) | 0.89(0.59,1.34) | 1.09(0.74,1.60) | 0.92 |  |
| Never | ref | 1.41(0.85,2.32) | 1.39(0.89,2.16) | 0.82(0.52,1.28) | 0.97 |  |
| **CVD** |  |  |  |  |  | 0.16 |
| Yes | ref | 1.22(0.83,1.80) | 0.89(0.60,1.32) | 0.87(0.57,1.33) | 0.2 |  |
| No | ref | 1.01(0.88,1.15) | 0.88(0.77,1.00) | 1.08(0.95,1.23) | 0.73 |  |
| **DM** |  |  |  |  |  | 0.52 |
| Yes | ref | 0.88(0.66,1.18) | 0.82(0.60,1.13) | 0.87(0.65,1.16) | 0.31 |  |
| No | ref | 1.05(0.90,1.21) | 0.90(0.78,1.04) | 1.11(0.97,1.28) | 0.55 |  |
| **Hypertension** |  |  |  |  |  | **<0.001** |
| Yes | ref | 0.96(0.79,1.16) | 0.67(0.55,0.81) | 0.69(0.55,0.87) | **<0.001** |  |
| No | ref | 1.01(0.85,1.19) | 0.97(0.82,1.15) | 1.30(1.11,1.52) | **0.41** |  |

Quartile 1: < 0.20 μg/L, Quartile 2: 0.21-0.35μg/L, Quartile 3: 0.36-0.65μg/L, Quartile 4: > 0.65μg/L

OSA: obstructive sleep apnea, DM: diabetes mellitus, CVD: cardiovascular disease, p-t, p for trend; p-int, p for interaction

| **Supplementary Table 4. Weighted logistic regression (OR and 95% CI) of sleep duration with blood cadmium modeling age, PIR, and BMI as categorical variables** | | | | | | | | |
| --- | --- | --- | --- | --- | --- | --- | --- | --- |
|  | **Sleep duration** | | | | | | | |
|  | Crude model | | Model 1 | | Model 2 | | Model 3 | |
| Cadmium | OR (95%CI) | P | 95%CI | P | OR (95%CI) | P | OR (95%CI) | P |
| **Normal sleep vs Insufficient sleep** | | | | | | | | |
| Quartile 1 | ref |  | ref |  | ref |  | ref |  |
| Quartile 2 | 0.93(0.75,1.23) | 0.85 | 1.21(0.89,1.44) | 0.70 | 1.01(0.82,1.28) | 0.57 | 0.67(0.47,1.06) | 0.68 |
| Quartile 3 | 1.05(0.92,1.26) | 0.90 | 1.19(0.85,1.45) | 0.06 | 1.07(0.93,1.25) | 0.36 | 0.78(0.55,1.21) | 0.52 |
| Quartile 4 | 1.62(1.23,1.81) | **<0.0001** | 1.67(1.28,1.90) | **<0.0001** | 1.33(1.11,1.58) | **<0.0001** | 1.15(0.99,1.35) | 0.45 |
| p for trend |  | **<0.0001** |  | **<0.0001** |  | **0.01** |  | 0.66 |
| LN cadmium | 1.32(1.20,1.50) | **<0.0001** | 1.50(1.35,1.67) | **<0.0001** | 1.39(1.25,1.55) | **0.01** | 1.38(1.19,1.86) | **0.01** |
| **Normal sleep vs Excessive sleep** | | | | | | | | |
| Quartile 1 | ref |  | ref |  | ref |  | ref |  |
| Quartile 2 | 0.73(0.54,1.17) | 0.25 | 0.64(0.44,1.18) | 0.30 | 0.83(0.66,1.65) | 0.32 | 0.71(0.54,0.89) | 0.06 |
| Quartile 3 | 1.03(0.84,1.14) | 0.88 | 0.52(0.34,1.15) | 0.34 | 0.63(0.39,1.64) | 0.43 | 0.55(0.37,1.54) | 0.44 |
| Quartile 4 | 1.24(1.12,1.67) | **0.02** | 1.56(1.10,1.96) | **0.05** | 0.90(0.67,1.22) | 0.50 | 0.82(0.52,1.36) | 0.33 |
| p for trend |  | **0.01** |  | **<0.0001** |  | 0.65 |  | 0.10 |
| LN cadmium | 1.52(0.71,2.06) | 0.10 | 1.34(0.98,1.82) | 0.07 | 1.00(0.56,1.37) | 0.29 | 0.68(0.41,1.45) | 0.59 |

Age was categorised into 20–39, 40–59, ≥ 60 years groups; BMI was categorised into <25, 25-30, ≥30 kg/m2 groups; family poverty-to-income ratio(PIR) was was categorised into <1, 1-3, ≥3 groups

Crudel model adjust for: none

Model 1 adjust for: age, gender, race

Model 2 adjust for: model 1 plus education level, marital status and family poverty-to-income ratio

Model 3 adjust for: model 2 plus cotinine level, BMI, smoking status, alcohol consumption, CVD, DM, Hypertension and general health

| **Supplementary Table 5. Weighted logistic regression (OR and 95% CI) of sleep-related disorders with blood cadmium modeling age, PIR, and BMI as categorical variables** | | | | | | | | |
| --- | --- | --- | --- | --- | --- | --- | --- | --- |
|  | Crude model |  | Model 1 |  | Model 2 |  | Model 3 |  |
| Cadmium | 95%CI | P | 95%CI | P | 95%CI | P | 95%CI | P |
| **Obstructive sleep apnea symptoms** | | | | | | | | |
| Quartile 1 | ref |  | ref |  | ref |  | ref |  |
| Quartile 2 | 1.01(0.80,1.12) | 0.71 | 1.05(0.92,1.20) | 0.44 | 1.11(0.88,1.39) | **0.01** | 0.76(0.51,1.03) | 0.62 |
| Quartile 3 | 0.77(0.59,1.22) | 0.06 | 0.92(0.80,1.07) | 0.28 | 0.93(0.66,1.14) | 0.26 | 0.79(0.65,1.16) | 0.12 |
| Quartile 4 | 1.49 (1.32, 1.59) | **<0.001** | 1.09(0.95,1.24) | 0.31 | 1.26(1.13,1.61) | **<0.001** | 1.45 (1.23, 1.68) | **<0.001** |
| p for trend |  | **<0.001** |  | **<0.001** |  | **<0.001** |  | **<0.001** |
| LN cadmium | 1.22(1.14,1.47) | **0.01** | 1.14 (1.02, 1.33) | **<0.001** | 1.40 (1.21, 1.52) | **<0.001** | 1.48 (1.35, 1.66) | **<0.001** |
| **Trouble sleeping** | | | | | | | | |
| Quartile 1 | ref |  | ref |  | ref |  | ref |  |
| Quartile 2 | 0.90(0.80,1.03) | 0.62 | 0.83(0.72,0.96) | 0.11 | 0.24(0.13,0.57) | 0.42 | 0.72(0.51,0.96) | **0.05** |
| Quartile 3 | 1.05(1.01,1.22) | 0.14 | 0.69(0.37,1.23) | 0.33 | 1.10(1.08,1.35) | **0.01** | 1.84(1.41,2.11) | **0.01** |
| Quartile 4 | 1.76(1.31,1.93) | **<0.001** | 1.01(0.82,1.12) | 0.41 | 1.23(1.12,1.64) | **<0.001** | 1.66(1.34,2.06) | **0.02** |
| p for trend |  | **<0.001** |  | **<0.001** |  | **<0.001** |  | **<0.001** |
| LN cadmium | 1.33(1.26,1.52) | **<0.001** | 1.22(1.18,1.38) | **<0.001** | 1.05( 1.02, 1.28) | **<0.001** | 1.64(1.12,2.13) | **0.01** |
| **Daytime sleepiness** | | | | | | | | |
| Quartile 1 | ref |  | ref |  | ref |  | ref |  |
| Quartile 2 | 0.78(0.62,1.17) | 0.09 | 0.65(0.52,1.22) | **0.02** | 0.77(0.72,1.20) | 0.45 | 1.15(1.01,1.30) | **0.04** |
| Quartile 3 | 0.94(0.82,1.09) | 0.07 | 0.97(0.84,1.13) | 0.71 | 1.15(1.02,1.44) | **0.01** | 0.94(0.78,1.17) | 0.36 |
| Quartile 4 | 1.24(1.16,1.44) | **<0.001** | 1.22(1.07,1.39) | **<0.001** | 1.35(0.98,1.38) | **<0.001** | 0.60(0.44,1.03) | 0.16 |
| p for trend |  | **<0.001** |  | **<0.001** |  | **0.01** |  | 0.06 |
| LN cadmium | 1.04(1.01,1.29) | **0.01** | 1.24(1.12,1.55) | **<0.001** | 1.28(1.13,1.55) | **0.01** | 0.82(0.66,1.02) | 0.12 |

Age was categorised into 20–39, 40–59, ≥ 60 years groups; BMI was categorised into <25, 25-30, ≥30 kg/m2 groups; family poverty-to-income ratio(PIR) was was categorised into <1, 1-3, ≥3 groups

Crudel model adjust for: none

Model 1 adjust for: age, gender, race

Model 2 adjust for: model 1 plus education level, marital status and family poverty-to-income ratio

Model 3 adjust for: model 2 plus cotinine level, BMI, smoking status, alcohol consumption, CVD, DM, Hypertension and general health

| **Supplementary Table 6. Unweighted characteristics of participants in the NHANES (2005–2008 and 2015–2020) by blood cadmium levels** | | | | | | |
| --- | --- | --- | --- | --- | --- | --- |
| **Variable** | **Total (n=15192)** | **Quartile 1 (n=3907)** | **Quartile 2 (n=3753)** | **Quartile 3 (n=3811)** | **Quartile 4 (n=3721)** | **P value** |
| **Age (years)** | 48.11±0.37 | 41.34±0.42 | 46.65±0.72 | 51.56±0.62 | 49.33±0.44 | < 0.0001 |
| **BMI (kg/m^2^)** | 29.31±0.13 | 29.45±0.52 | 29.12±0.15 | 28.37±0.34 | 27.85±0.21 | < 0.0001 |
| **Family poverty-to-income ratio** | 3.09±0.03 | 3.279±0.04 | 3.36±0.06 | 3.05±0.05 | 2.61±0.03 | < 0.0001 |
| **Cotinine (ng/mL)** | 60.54±2.32 | 17.67±2.04 | 18.63±2.21 | 32.97±2.31 | 190.75±5.08 | < 0.0001 |
| **Gender** |  |  |  |  |  | < 0.0001 |
| Female | 7573(50.80) | 1512(38.12) | 1913(52.73) | 2252(62.92) | 1896(52.99) |  |
| Male | 7619(49.20) | 2395(61.88) | 1840(47.27) | 1559(37.08) | 1825(47.01) |  |
| **Race/ethnicity** |  |  |  |  |  | < 0.0001 |
| Mexican American | 2393(7.88) | 709(9.17) | 732(9.49) | 602(7.73) | 350(4.54) |  |
| Non-Hispanic black | 3304(10.12) | 773( 8.23) | 750( 9.52) | 829(10.79) | 952(12.62) |  |
| Non-Hispanic white | 6732(70.32) | 1740(72.76) | 1576(68.93) | 1650(68.41) | 1766(70.63) |  |
| Others | 2763(11.67) | 685( 9.84) | 695(12.07) | 730(13.07) | 653(12.21) |  |
| **Education level** |  |  |  |  |  | < 0.0001 |
| Completed high school | 3572(24.29) | 817(20.95) | 786(20.74) | 892(25.21) | 1077(31.74) |  |
| Less than high school | 3471(14.71) | 696( 9.41) | 777(12.64) | 911(15.48) | 1087(23.22) |  |
| More than high school | 8149(61.00) | 2394(69.64) | 2190(66.62) | 2008(59.31) | 1557(45.04) |  |
| **Marital status** |  |  |  |  |  | < 0.0001 |
| Married/living with partner | 9242(64.92) | 2463(65.46) | 2427(68.66) | 2350(67.10) | 2002(57.78) |  |
| Never married | 2628(17.00) | 910(23.53) | 599(14.98) | 498(12.10) | 621(15.66) |  |
| Widowed/divorced | 3322(18.09) | 534(11.02) | 727(16.36) | 963(20.80) | 1098(26.57) |  |
| **Smoking status** |  |  |  |  |  | < 0.0001 |
| Former | 3747(25.29) | 779(21.34) | 1018(28.86) | 1283(34.61) | 667(16.98) |  |
| Never | 8303(54.09) | 3043(76.47) | 2558(66.18) | 2026(50.57) | 676(14.64) |  |
| Now | 3142(20.63) | 85( 2.19) | 177( 4.95) | 502(14.82) | 2378(68.37) |  |
| **Alcohol consumption** |  |  |  |  |  | < 0.0001 |
| Current heavier drinker | 3153(21.94) | 819(21.27) | 667(18.37) | 617(17.64) | 1050(31.19) |  |
| Current light/moderate drinker | 8032(55.84) | 2244(60.68) | 2047(58.76) | 2010(56.11) | 1731(45.92) |  |
| Former | 2039(12.17) | 354( 7.56) | 502(12.14) | 591(14.19) | 592(16.19) |  |
| Never | 1968(10.06) | 490(10.49) | 537(10.73) | 593(12.06) | 348( 6.70) |  |
| CVD |  |  |  |  |  | < 0.0001 |
| No | 13541(91.85) | 3642(95.25) | 3406(92.90) | 3318(89.75) | 3175(88.32) |  |
| Yes | 1651( 8.15) | 265( 4.75) | 347( 7.10) | 493(10.25) | 546(11.68) |  |
| DM |  |  |  |  |  | **0.04** |
| No | 12388(86.34) | 3225(87.56) | 3020(85.16) | 3065(85.50) | 3078(86.91) |  |
| Yes | 2804(13.66) | 682(12.44) | 733(14.84) | 746(14.50) | 643(13.09) |  |
| Hypertension |  |  |  |  |  | < 0.0001 |
| No | 9075(64.78) | 2617(71.14) | 2266(65.45) | 2113(59.88) | 2079(60.67) |  |
| Yes | 6117(35.22) | 1290(28.86) | 1487(34.55) | 1698(40.12) | 1642(39.33) |  |
| **General health** |  |  |  |  |  | < 0.0001 |
| Excellent | 2019(16.20) | 558(16.98) | 555(18.95) | 552(17.25) | 354(11.00) |  |
| Very good | 4223(32.82) | 1211(36.53) | 1078(34.49) | 1045(31.85) | 889(27.05) |  |
| Good | 5489(34.85) | 1397(33.88) | 1349(32.79) | 1359(34.57) | 1384(38.73) |  |
| Fair | 2851(13.46) | 634(11.02) | 643(11.75) | 722(13.91) | 852(18.13) |  |
| Poor | 610( 2.67) | 107(1.58) | 128(2.02) | 133(2.43) | 242(5.09) |  |
| **Sleep duration** |  |  |  |  |  | < 0.0001 |
| Excessive sleep | 966( 4.86) | 240(4.85) | 207(4.06) | 248(4.81) | 271(5.83) |  |
| Insufficient sleep | 4945(30.81) | 1194(28.78) | 1191(28.75) | 1191(29.13) | 1369(37.50) |  |
| Normal sleep | 9281(64.33) | 2473(66.37) | 2355(67.19) | 2372(66.06) | 2081(56.67) |  |
| **OSA symptoms** |  |  |  |  |  | **0.01** |
| No | 10629(69.82) | 2901(64.26) | 2596(69.18) | 2735(72.03) | 2397(64.42) |  |
| Yes | 4563(30.18) | 1006(25.74) | 1157(30.82) | 1076(27.97) | 1324(35.58) |  |
| **Daytime sleepiness** |  |  |  |  |  | **0.02** |
| No | 11984(77.91) | 3153(80.70) | 2977(79.01) | 3058(78.75) | 2796(75.14) |  |
| Yes | 3208(22.09) | 754(19.30) | 776(20.99) | 753(21.25) | 925(24.86) |  |
| **Trouble sleeping** |  |  |  |  |  | < 0.001 |
| No | 11301(72.87) | 2950(74.31) | 2856(75.11) | 2855(71.63) | 2640(69.73) |  |
| Yes | 3891(27.13) | 957(25.69) | 897(24.89) | 956(28.37) | 1081(30.27) |  |

Quartile 1: < 0.20 μg/L, Quartile 2: 0.21-0.35μg/L, Quartile 3: 0.36-0.65μg/L, Quartile 4: > 0.65μg/L

NHANES, National Health and Nutrition Examination Survey, BMI: body mass index , DM: diabetes mellitus, CVD: cardiovascular disease, OSA: obstructive sleep apnea
